# The assembly and importance of a novel ecosystem: The ant community of coffee farms in Puerto Rico

**DOI:** 10.1002/ece3.6785

**Published:** 2020-10-27

**Authors:** Ivette Perfecto, John Vandermeer

**Affiliations:** ^1^ School for Environment and Sustainability University of Michigan Ann Arbor MI USA; ^2^ Department of Ecology and Evolutionary Biology University of Michigan Ann Arbor MI USA

**Keywords:** biological control, coexistence, community structure, dominance, invasive species, *Solenopsis invicta*, *Wasmannia auropunctata*

## Abstract

Agricultural ecosystems are by their very nature novel and by definition the more general biodiversity associated with them must likewise constitute a novel community. Here, we examine the community of arboreally foraging ants in the coffee agroecosystem of Puerto Rico. We surveyed 20 coffee plants in 25 farms three times in a period of one year. We also conducted a more spatially explicit sampling in two of the farms and conducted a species interaction study between the two most abundant species, *Wasmannia auropunctata* and *Solenopsis invicta,* in the laboratory. We find that the majority of the most common species are well‐known invasive ants and that there is a highly variable pattern of dominance that varies considerably over the main coffee producing region of Puerto Rico, suggesting an unusual modality of community structure. The distribution pattern of the two most common species, *W. auropunctata* and *S. invicta*, suggests strong competitive exclusion. However, they also have opposite relationships with the percent of shade cover, with *W. auropunctata* showing a positive relationship with shade, while *S. invicta* has a negative relationship. The spatial distribution of these two dominant species in the two more intensively studied farms suggests that young colonies of *S. invicta* can displace *W. auropunctata*. Laboratory experiments confirm this. In addition to the elaboration of the nature and extent of this novel ant community, we speculate on the possibilities of its active inclusion as part of a biological control system dealing with several coffee pests, including one of the ants itself, *W. auropunctata*.

## Graphical abstract

Here, we describe the arboreally foraging ant community in 25 coffee farms in Puerto Rico. We find that the majority of the dominant species are non‐native. Through a detailed study of two of the farms, we describe the interactions between two non‐native dominant species, *Wasmannia auropunctata* and *Solenopsis invicta*.

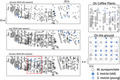

## 1 INTRODUCTION

Novel ecosystems present an opportunity that has been common yet rarely recognized in the field of ecology; the opportunity to study how “not‐necessarily‐coevolved” organisms come together and structure an ecological community (Evers et al., 2018; Godoy, 2019; Hobbs et al., 2006; Hobbs, Higgs, & Harris, 2009; Perfecto & Vandermeer, 2015). Our ability to understand these new systems is a test of the extent to which we understand the natural laws that determine community and ecosystem structure (Perfecto & Vandermeer, 2015). Ants represent an interesting case in that they form novel communities that are consistent both taxonomically (all species in the same clade) and ecologically (all species live in similar ecological niches). There are some functional and phylogenetically distinguishable categories, to be sure. For example, the division into carbohydrate‐dependent species versus protein‐dependent species (Davidson, 1997) imperfectly but sensibly partitions species according to a niche trait, and the four subfamilies, Myrmecinae, Pseudomyrmecinae, Formicinae, and Dolichodorinae, make phylogenic sense of a plethora of species with a similar range of ecological niches. Although the Poneriniformes present both ecological and phylogenetic problems (Ward, 2007), and the Dorylinae combine monophylogeny with the obvious ecological niche of predator with special behavior, the four main subfamilies contain species whose niches are relatively consistent. Here, we are mainly concerned with these more generalized four omnivore subfamilies, which include most of the world's most infamous invasive ant species.

Community structure is a complicated subject even when restricted to a small guild operating on a single trophic level, as is the case here. Yet the very novelty of the system provides a unique view of how the various components fit together. For example, while competitive exclusion is expected to permit only minimal niche overlap among coexisting species, such an expectation is not palpable in the novel ecosystem context since the tacit assumption of equilibrium is rarely justified. Habitat specialization at various scales is frequently thought to account for many coexistence patterns, certainly a key factor in ant communities. Migration and extinction patterns represent a distinct level of explanatory phenomena, undoubtedly of potential importance in a spatially distributed system which is the case in the present study. The important issue of “invasion meltdown” (Simberloff, 2006), in which non‐native species facilitate one another's invasion, is frequently cited in warnings of the impact of invasive species. In contrast, the eventual reduction of the impact of a key invasive species, almost the inverse of the invasion meltdown idea, is frequently noted (Braga, Gómez‐Aparicio, Heger, Vitule, & Jeschke, 2018; Lach & Hooper‐Bui, 2010). Both invasion meltdown and impact reduction strongly suggest that transient phenomena rather than equilibria are dominant, in terms of all elements of community dynamics, including population densities, species compositions, and species interactions.

In a series of important studies, Torres (1984a, 1984b) summarized much of the knowledge obtainable from the distribution of ants on the island of Puerto Rico, concentrating on ecological observables such as food type, habitat occurrence, island isolation, and microhabitat factors. Here, we effectively restrict our analysis to one particular habitat type, the coffee agroecosystem, with its community of mainly non‐native ants, clearly within the general category of a novel ecosystem (Perfecto & Vandermeer, 2015). It is worth noting that ants present a particularly interesting problem from a practical point of view. On the one hand, they are most frequently generalist predators (Eubanks, 2001; Offenberg, 2015; Perfecto & Castiñeiras, 1998; Philpott & Armbrecht, 2006; Philpott, Perfecto, Armbrecht, & Parr, 2010; Torres & Snelling, 1997) and thus of potential importance in providing the ecosystem service of pest control. On the other hand, some species are regarded as noxious pests themselves, with leaf‐cutting ants reducing photosynthetic area, mutualists protecting hemipteran herbivores, or fire ants stinging farmers and farm workers (Fabres & Brown, 1978; Haines & Haines, 1978; Jetter, Hamilton, & Klotz, 2002; Reimer, Beardsley, & Jahn, 1990). Understanding how the overall community of ants is structured thus has important practical implications, in addition to the more theoretical justification of understanding community structure through the lens of the novel ecosystem.

In this study, we take the opportunity to study how “not‐necessarily‐coevolved” organisms come together to form an ecological community, using the “novel ecosystem” of ants on coffee farms as a focal system. The background habitat is easily recognized as “the coffee system” which presents an environmental background that has both consistency (all sites are coffee farms, and all are in the central mountain range of Puerto Rico) and variability (management styles vary from farm to farm). The consistency is more notable than the variability under casual observation, and we can presume that the general population and community dynamics of the organisms making up the novel ecosystem are the main drivers of community assembly. What that assembly looks like, qualitatively, is the underlying goal of this study.

In focusing on this particular community, we find unsurprisingly, that there is an unequal distribution among species, more rare species than common ones at a given site. Most evident in this situation is the occurrence of two particular species, *Solenopsis invicta* and *Wasmannia auropunctata*, both of which are non‐native and happen to be regarded as pests by local farmers. Given the commonness of these two species, and given the obvious observation that they rarely occurred together as common occupants on any given farm, it was most natural to focus on them as an important dynamic component of the overall community structure. Thus, much of this study focuses on these two species as an important element of the overall community dynamics.

## 2 METHODS

**Figure 1:**
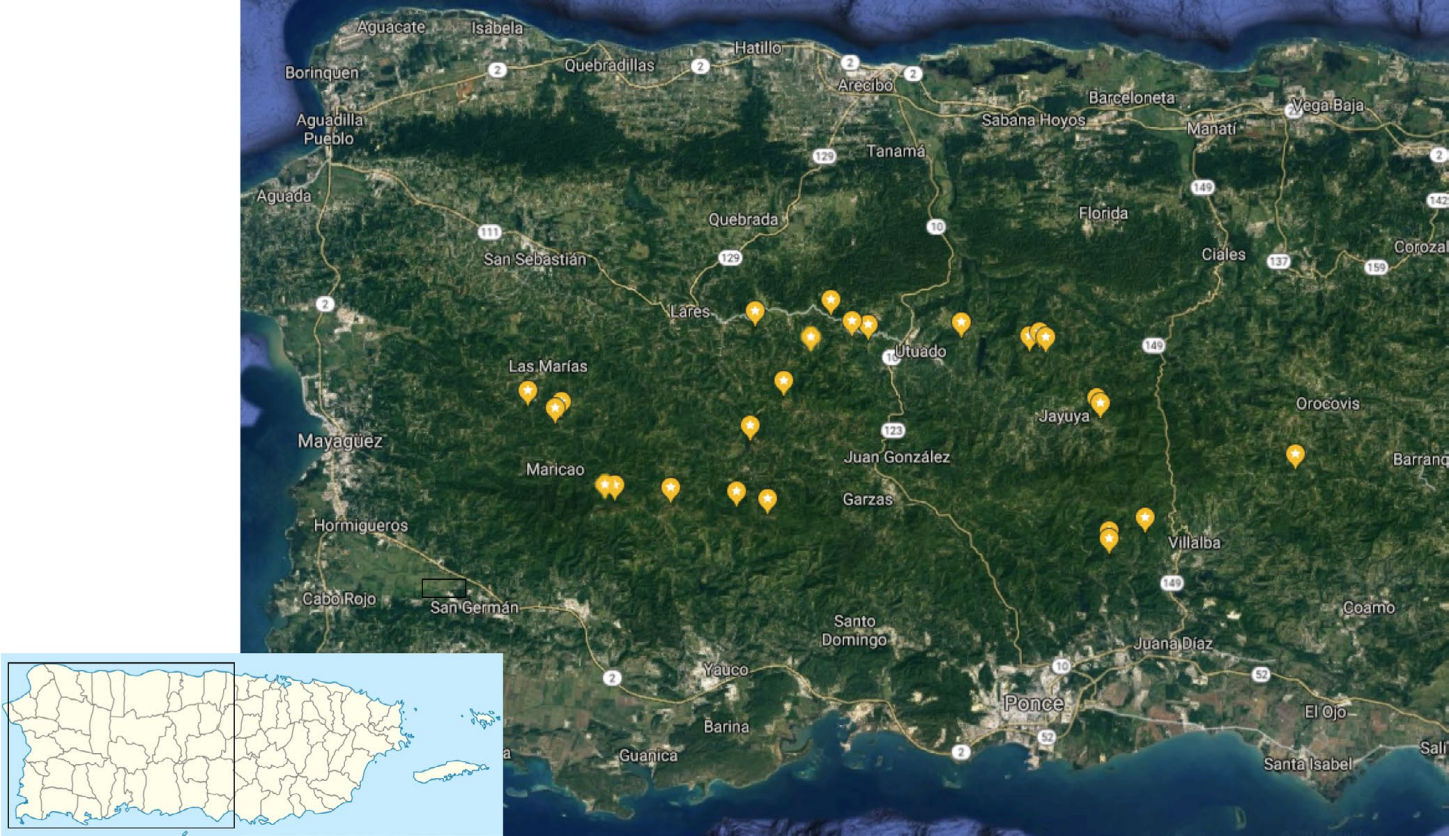
Positions of the 25 farms in the sample. Image from Google Earth

From a survey of 85 coffee farms throughout the coffee‐growing region of Puerto Rico (effectively from the municipality of Orocovis to Las Marias), we chose 25 as representative of the habitat types, based on shade cover and geographic position. That is, we chose the farms to study based on an intention to sample the whole range of coffee farms on the island. The position of all farms studied is shown in Figure 1, and the basic geographic information (latitude, longitude, and elevation) and percent canopy cover can be found in Table S1 in the supplementary material. Farms were separated from each other by a minimum distance of one kilometer, but most farms were separated by more than 5 km. Since the area is relatively small, climatic conditions vary little across the farms, with the ones located further south (subtropical moist forest: 1,000–2,000 ml annual precipitation) being drier than the ones further north (subtropical wet forest: 2,000–4,000 ml annual precipitation), and the northern ones being closer to the massive limestone formations (known locally as mogotes) on the north west side of the island (Miller & Lugo, 2009). It is unlikely that any of these geographic conditions affect the ant communities, and our results offer no hint that such could be the case.

The study farms were located over the whole range of the coffee‐growing area and included farms that ranged from very sunny (low shade cover) to highly shaded (Table S1). Preliminary analysis of these habitat factors demonstrated no relationship between management type or geographic position and the ant community, so these variables are not pursued further in this study.

During the months of December 2018 and January 2019, we visited each of the farms and placed five tuna fish baits directly on the stem (or stems) of each of 20 coffee plants (baits stuck easily on the bark of the plant), chosen randomly from a 10 × 10m plot, which, in turn was chosen to reflect the basic management style of the farm. Thus, we placed a total of 100 arboreal baits in a representative area of 100 m^2^ on each of 25 farms, waited for 40 min and checked each bait for ants, recording presence (no counts of numbers of foragers) at each bait. Since the number of foragers on a bait is more an indication of the activity of a nearby nest and has very little to do with the abundance of the species itself, it is wise to reject any notion of counting workers as some sensible indication of population density or abundance. More relevant is simply the number of baits occupied, in the present case the number of baits occupied on a given tree ranged from 0 to 5, meaning that our estimates of abundance on a given tree always ranges from 0 to 5. Most species were identified in the field, and specimens collected and examined in the laboratory only for those cases when the identity was not obvious. On each farm, the species that occupied the most coffee trees was called the dominant species. It was almost always the case that one species was clearly dominant in this sense, although in a few cases two or three species were almost equally represented with respect to number of trees occupied and, in a few cases, there was no clear dominance. Subdominant is defined as occurrence on <10 observations over the course of the study, on a particular farm. All farms were revisited in July 2019 and January 2020, and the sampling was repeated using the same methodology. Based on the work of Tschinkel (1988), we noted that it was almost always possible to distinguish two basic forms of swarms of the red imported fire ant, *Solenopsis invicta* on the baits, one form with almost all individuals of small or “minor” proportions and the other form with a few to many very large or “major” forms, especially noted for a very large gaster. Based on Tschinkel's results, we interpreted these two forms as “young” colonies versus “old” colonies, since it seems that in younger colonies, the queens produce only minor workers and only when they reach an older age do they begin producing what seems to be a totally different cast of individuals, the majors. We also noticed that the characteristic sting of *S. invicta* with the formation of an evident pustule on the skin at the site of the sting seems to be caused only by these major workers. Furthermore, the most common phorid fly parasitoid observed in all our *S. invicta* samples seemed to strongly prefer attacking the major workers, as has been reported elsewhere (Williams & Banks, 1987). The information on worker size was used to help interpret some of our findings as reported in the results.

It is evident from our 25 farm surveys that the most dominant ants are also the ones frequently cited by farmers as undesirable because of their potent stings, *Wasmannia auropunctata* and *S. invicta*, although these two species are also potentially important as providers of the ecosystem service of pest control (Eubanks, 2001; Morris, Jimenez‐Soto, Philpott, & Perfecto, 2018; Morris & Perfecto, 2016). Especially important is the locally named *abyarde* (electric fire ant), *W. auropunctata*, which occurs in large patches on the farms and is such a nuisance to workers during the harvest that efficiency of harvest is dramatically reduced since workers tend to skip areas that have concentrations of this species (I. P. personal communication with farmers in Puerto Rico). Two farms were chosen for more detailed study of these two species (codes for all farms are listed in Table S1 in the supplementary material, also see caption to Table 2), *W. auropunctata and S. invicta,* at a larger scale, UTUA 2 (Finca Gran Batey) and UTUA 20 (Finca Cítricos, Inc), the first dominated by *W. auropunctata* and the second by *S. invicta* in the 10 × 10m plots located on those farms. On those two farms, we geolocated all coffee bushes (550 bushes in UTUA2 and 479 on UTUA 20) on an area of 2,500 m^2^ in UTUA 2 and on an area of 1,950 m^2^ in UTUA 20, placed five baits on each coffee plants, let the baits set for 40 min and then recorded the ant species on each of them. We sampled on these two farms once in December/January 2018/2019, once in July 2019 and once in January 2020, effectively covering a twelve‐month period. Sampling of the larger areas was limited by roads, fences, and other limitations of the section of the farm we sampled.

As described in the results, our third sampling time in the UTUA 2 farm revealed what appeared to be an invasion of the area previously dominated by *W. auropunctata* by swarms of young *S. invicta*. From many natural history observations, we understand that some of the competitive interactions between these two species take place on the ground. Recognizing that part of the expected competitive interactions of these two species occurs not only on coffee bushes but also on the ground and in the citrus trees above the coffee, we sampled these two venues as well in January 2020. Placing five tuna baits on each of the citrus trees within a 25 × 25 m^2^ area, we examined each bait for the occurrence of all ants after a 40 min waiting period. Within the same 25 × 25 m^2^ plot, we set up a 4 × 4 m grid on the ground and placed baits to sample ants on the ground. This plot was located in a section of the area where we discovered the apparent local invasion of *S. invicta*.

Finally, we performed six interaction trials between *S. invicta* and *W. auropunctata* in the laboratory. Fractions of nests of *S. invicta* and *W. auropunctata* containing workers and brood were introduced into nesting boxes (15 × 15 × 15 cm plastic containers), augmented with water and honey. After 5 days, nesting boxes were connected with pipe cleaners, and behavioral observations made. A week later, all nests were harvested, and number of workers estimated in all 12 containers (six interspecific comparisons).

## 3 RESULTS

### 3.1 10 × 10 m^2^ surveys in 25 farms: species richness and dominance

**Figure 2:**
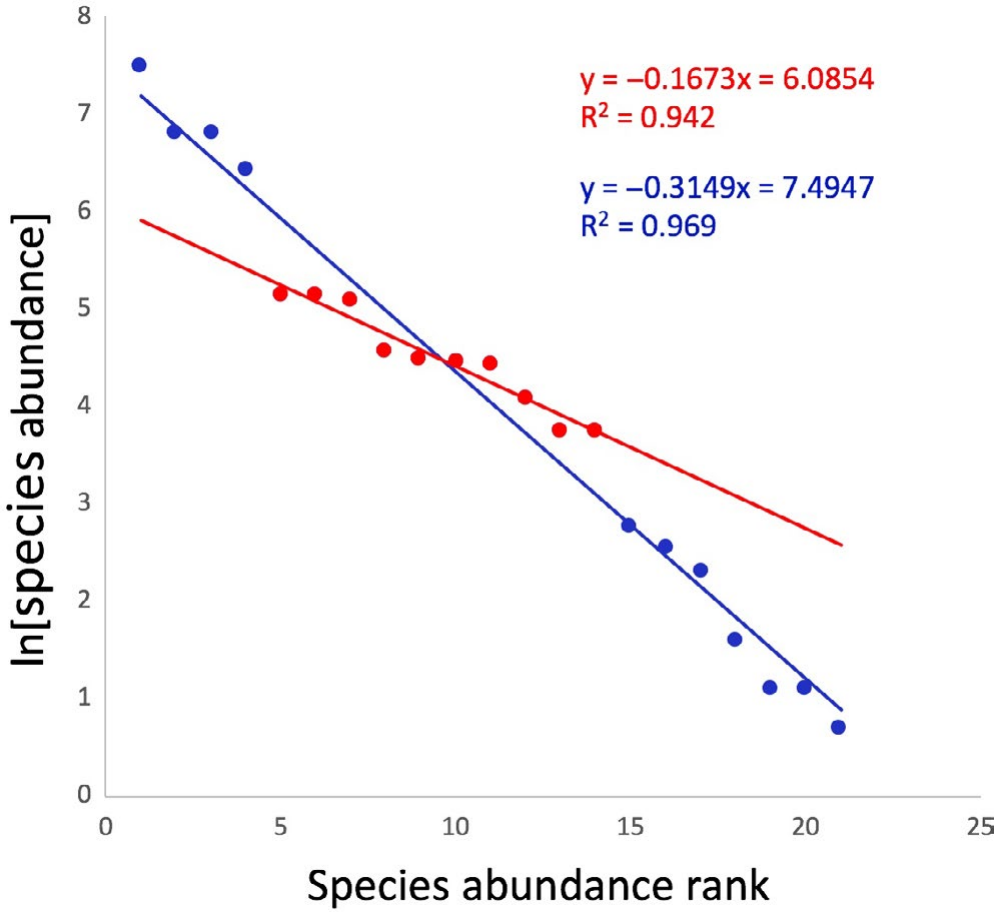
Power function relationship between species abundance and species rank. Complete collection consists of all point with a subset of the intermediate ranks in red. The four most abundance species seem somewhat out of the general pattern for the intermediate ones (in red with the shallower slope)

A total of 21 species (and/or morphospecies) of ants were recorded in the study (Table 1). Eight were dominant on one or more (but never all) of the farms, while others were very active but only rarely dominant (Table 2). Collating all the species together from all sites over all three years, the pattern of number of species versus rank abundance follows the typical power law, known in ecology since at least the 1940s (Fisher, Corbet, & Williams, 1943) (Figure 2). This regularity is frequently interrogated from the point of view of underlying mechanism (e.g., Hubbell, 2001), a research program reflected in our unaggregated data, as presented in Table 1 and further explored below. The linear relationship between the natural log of species abundance (number of bushes on which the species occurred) and the rank of the species (most abundant first, least abundance last) has been argued to be one of the most important fundamental tools in community ecology due to the universality of the pattern produced and the insights it provides about how communities are organized (MacArthur, 1957; McGill et al., 2007).

**Table 1:** List of species encountered and basic distributional statistics

Ant Species	Number of times/farms dominant	Total abundance	Number of times/farms occurrence	Presumed origin
*Wasmannia auropunctata*	25	1776	48	American Tropics (not PR)
*Solenopsis invicta*	14	919	65	South America
*Monomorium floricola*	13	917	41	South Asia
*Tapinoma melanocephala*	7	619	38	Old World Tropics
*Brachymyrmex heeri*	0	205	42	Native
*Linepithema iniquum*	3	187	13	Native
*Monomorium ebenium*	1	171	13	Native
*Tetramorium bicarinatum*	2	98	2	Southeast Asia
*Nylanderia pubens*	0	89	16	Native
*Pheidole megacephala*	0	88	20	Africa
*Nylanderia fulva*	0	69	3	South America
*Brachymyrmex obscurior*	0	59	19	Native
*Cardiocondyla emeryi*	1	43	3	Native
*Myrmelachista remulorum*	0	29	5	Native
*Paratrichina longicornis*	0	23	6	Native
*Pheidole moerens*	0	16	5	Native
*Pheidole exigua*	0	10	2	Native
*Pheidole sculptior*	0	5	2	Native
*Solenopsis* sp. 1	0	3	2	Not known
*Solenopsis* sp. 2	0	3	3	Not known
*Cardiocondyla venustula*	0	2	2	Africa

**Table 2:** Farms and dominant species on all three sampling dates (January 2019 [covering a sampling period from December 2018 to January 2019], July 2019 and January 2020)

Site Code	January 19	July 19	January 20	Species Identification
UTUA16	W	W	W	W = *Wasmannia auropunctata*
UTUA 2	W	W	W	S = *Solenopsis invicta*
MARI3	W	W	W	Tm = *Tapinoma melanocephala*
LASM3	W	W	W	Mf = *Monomorium floricola*
LASM1	W	W	W	L = *Linepithema iniquum*
OROC1	W	W	W	N = *Nylanderia fulva*
UTUA10	Tm	Tm	Tm	Tb = *Tetramorium bicarinatum*
UTUA20	S	S	S	C = *Cardiocondyla emeryi*
YAUC3	S	S	S	Me = *Monomorium ebenium*
UTUA30	Mf	Mf	Mf	ND = No Dominance
ADJU7	Mf	Mf	Mf	
JUAN7	Mf	Mf	Mf	
UTUA18	L	L	L	
MARI2	W	W	C	
JUAN1	W	Tm	Tm	
PONC1	W	S	S	
MARI18	W	N	W	
JAYU3	Tm	Mf/Tm	Mf	
UTUA17	Tm	Mf	Mf	
UTUA5	S	W/Mf/Tm	W/Mf/Tm	
ADJU8	S	S	Mf	
LASM2	S	Me	W	
UTUA13	S	Mf	S	
JAYU2	ND	Tb	Tb	
YAUC4	ND	ND	ND	

Note

Farm code indicates municipality and farm number code within the municipality (code numbers stem from previous larger sample of coffee farms).

Abbreviations: ADJU, Adjuntas; JAYU, Jayuya; JUAN, Juana Días; LASM, Las Marias; MARI, Maricao; OROC, Orocovis; UTUA, Utuado; YAUC, Yauco.

Over our whole sampling region (which was designed to sample the entire background habitat in which the dominant understory species is coffee), temporal consistency of the dominant ant species was variable (Table 2). Of the 25 farms, 13 were consistent with the same dominant species on all three sampling dates. Of the 12 farms that experienced a change in the dominant species, two of them had major activity by two invasive species, *Tetramorium bicarinatum* and *Nylanderia fulva*, neither species of which was encountered on any of the farms on the first sampling date, nor any other farms on the second sampling date, but were extremely common on the farms where they occurred.

**Figure 3:**
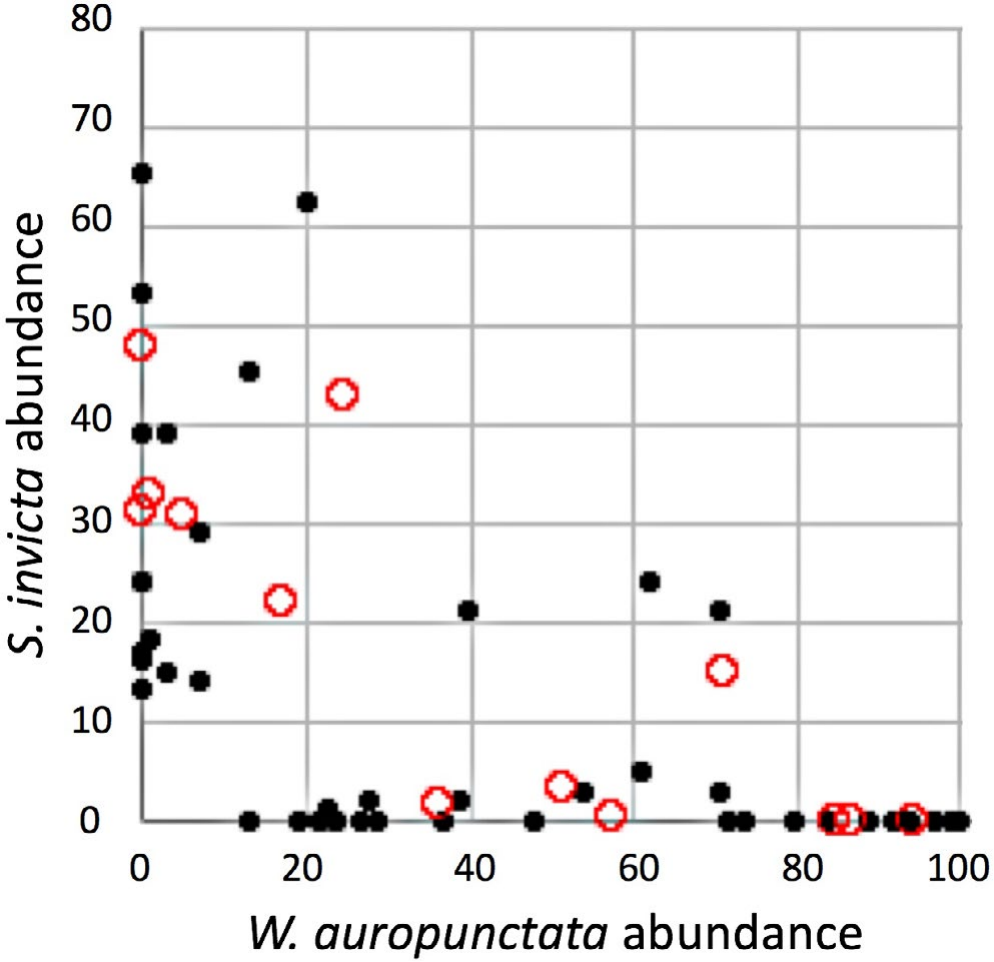
Abundance at a site of the two most common species by visit (black closed circles) or by average of three visits at a site (open red circles). Note the strong tendency of one or the other being dominant, with only four visits exhibiting more than 20 (out of 100) bait occupancies of both species

**Figure 4:**
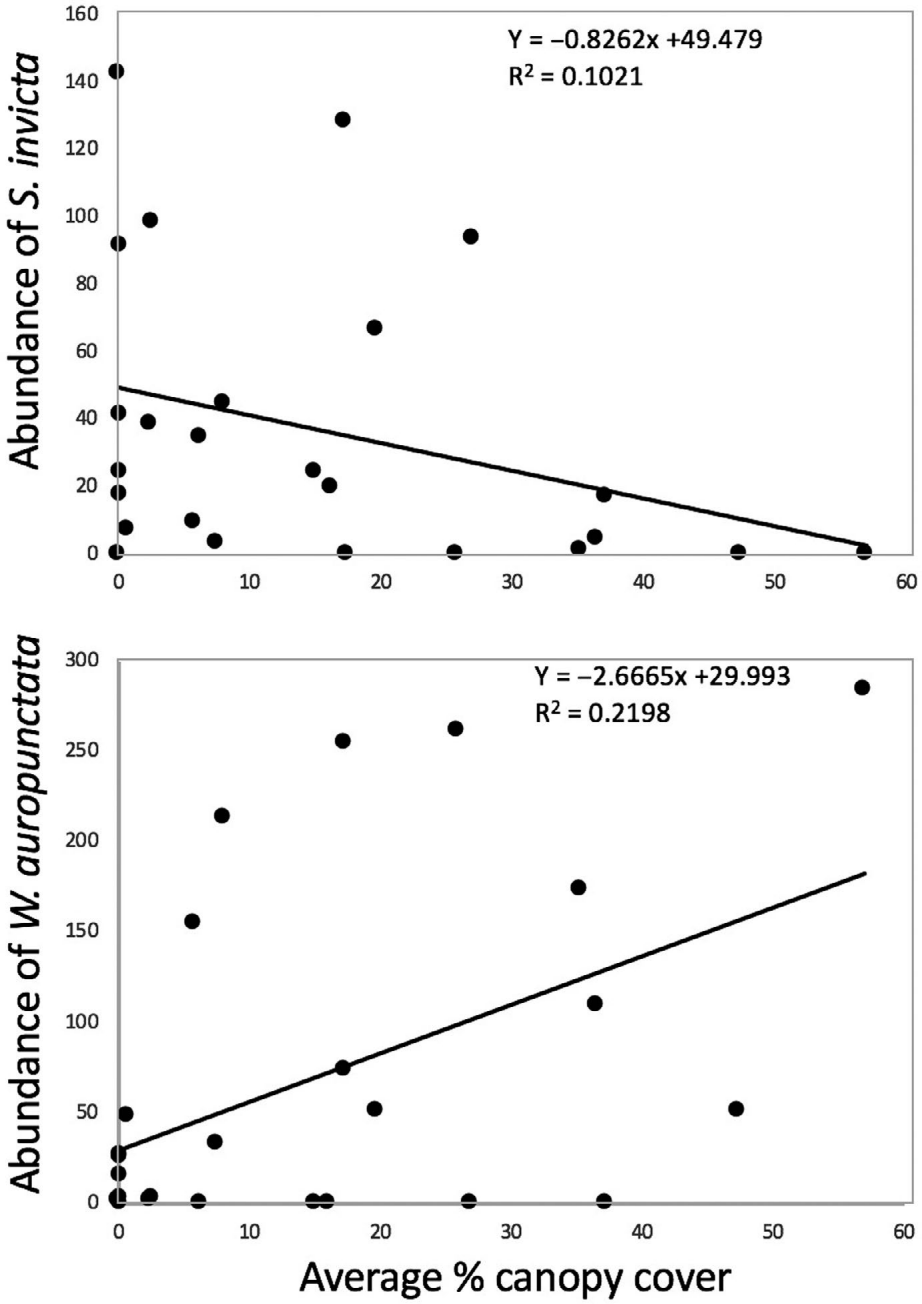
Relationship between the two most common species and average percent of shade cover

It is notable that from our 25 farm surveys we find that two of the most dominant ants are also the ones frequently cited by farmers as undesirable because of their potent stings (*W. auropunctata* and *S. invicta*), as mentioned above. It is also evident that these two species are the most common species (Table 1), although some farms had very low activity of either. Eliminating those site visits that had fewer than 10 individuals of either/or *W. auropunctata* or *S. invicta*, the abundance of the two is plotted in Figure 3. There is, for the most part, a dominance of one or the other of these two species. In all 75 farm surveys (25 farms surveyed three times), in only 11 surveys did we fail to find one or the other, and in the remaining 64 surveys, one or the other was clearly subdominant (observed less than 10 times) in all but five surveys. Thus, in consideration of these two species only, in almost 80% of the cases, there was clear dominance of one or the other (Figure 3), a pattern consistent with a strong competitive exclusion of one by the other, not necessarily in one direction or the other. Of course, such data are also consistent with a hypothesis of some underlying habitat factor that might be causing the pattern, especially the amount of shade in the system, a factor well‐known to influence ant abundance in the coffee system (Armbrecht & Gallego, 2007; Pardee & Philpott, 2011; Philpott et al., 2010; Teodoro, Sousa‐Souto, Klein, & Tscharntke, 2010). While there is no geographical pattern associated with dominance of either of these two species, and in three of the farms there was a change in the dominance of one to the other, there was a clear relationship between the average canopy cover and abundance (number of baits occupied), for both *W. auropunctata* and *S. invicta* (Figure 4).

### 3.2 Spatial distribution of the two dominant species in two farms

**Figure 5:**
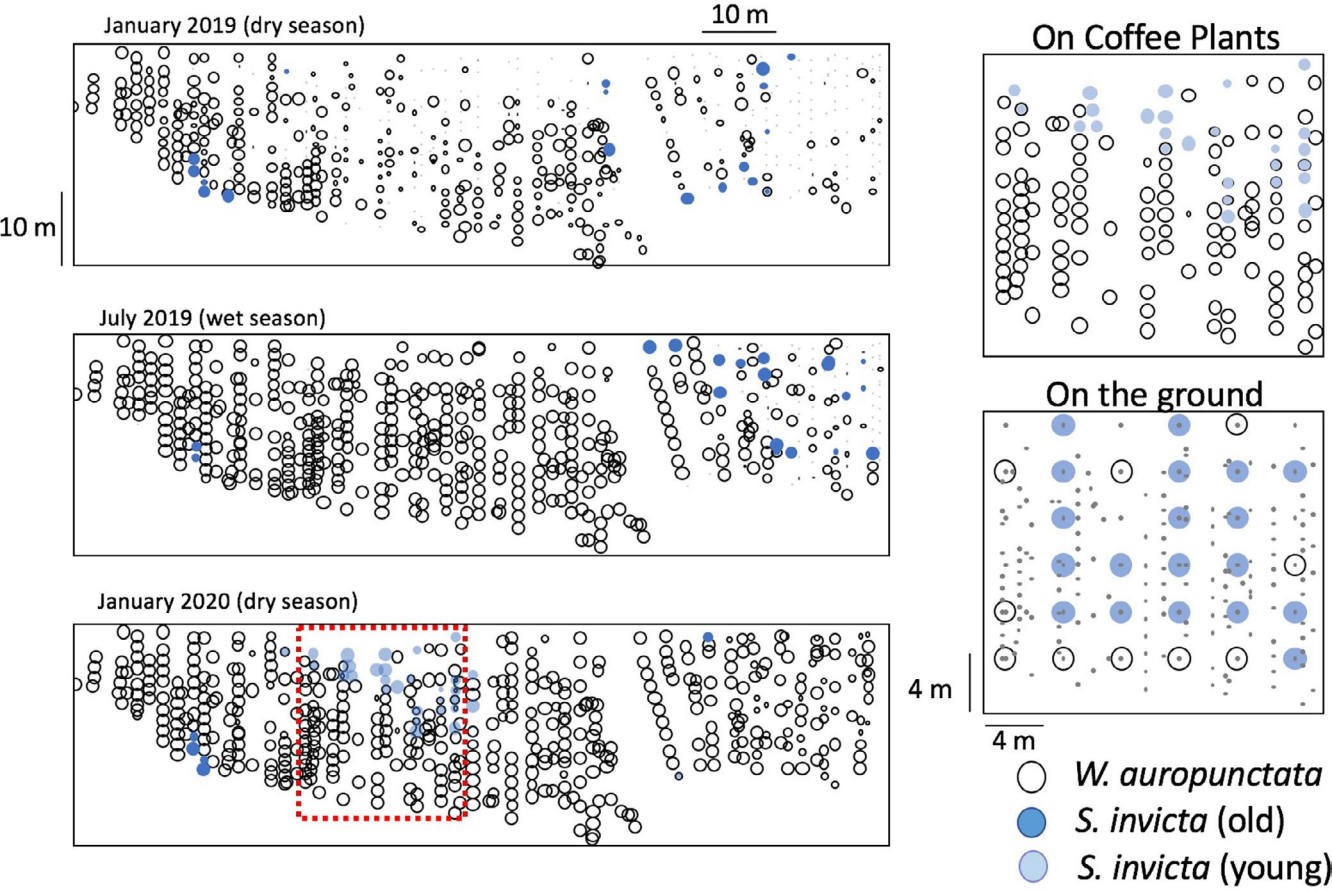
A 12‐month record of the spatial dynamics of two of the most dominant species on farm UTUA 2 (grid is 5 × 5 m^2^). Size of the symbol proportional to activity at that site (largest = 5 baits occupied, smallest = 1 bait occupied, small dots are bushes that had no individuals of the three species on any of the five baits). Dashed outline square in January 2020(dry) frame indicates the position of area baited with ground baits and presented on the right. All ground baits with *S. invicta*, where apparently from young colonies

**Figure 6:**
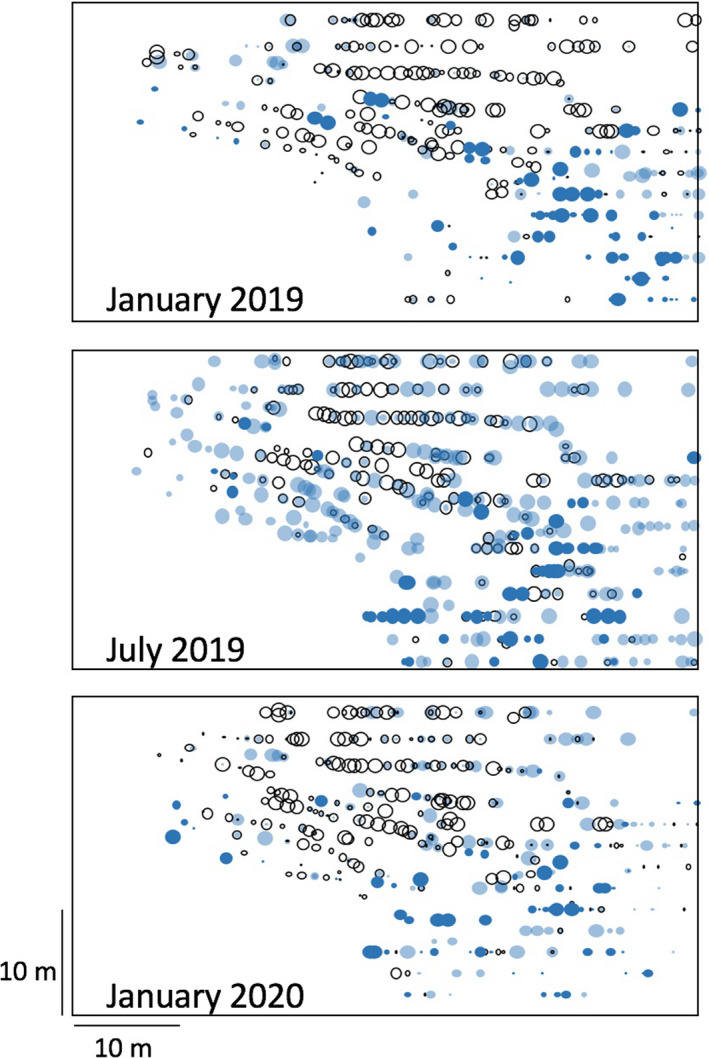
A 12‐month record of the spatial dynamics of two of the most dominant species on farm UTUA 20

In Figures 5 and 6, we display the results of the larger areas sampled on farms UTUA 2 and UTUA 20 for the two dominant species, *W. auropunctata* and *S. invicta*. There are clear patterns on these two farms over the 12‐month interval. In UTUA 2, the dominance of *W. auropunctata* increased between January 2019 and July 2019, but there was also an expansion of *S. invicta* into the area where *W. auropunctata* had been rare (the right side of the sampling area), although *W. auropunctata* had increased there also (Figure 5). That expansion of *W. auropunctata* continued on the right section of the plot between July 2019 and January 2020, but, notably, there also appeared a cluster of coffee bushes that were dominated by *S. invicta*. Interestingly, these new *S. invicta* bushes were all classified as from young colonies (based on the absence of very large workers, as explained in the methods section). For closer examination of the region in which these young swarms were evident, we set ground tuna baits at 4m intervals on a 20x20m grid and found that the “incursion” of *S. invicta* into the region formerly dominated by *W. auropunctata*, was considerably larger than evidenced in the observations strictly on coffee bushes, suggesting that this new “incursion” of *S. invicta* into the area previously dominated by *W. auropunctata* was driven by terrestrial (ground) dynamics involving these two species (Figure 5). The pattern might suggest that the presence of *S. invicta* is limiting the further expansion of *W. auropunctata*, although the mechanism driving this limitation remains obscure (as discussed further below). All but one of the 12 citrus trees sampled were dominated by *W. auropunctata*, suggesting that the displacement of this species by *S. invicta* starts with the establishment of *S. invicta* on the ground followed by foraging on coffee bushes, but not on the citrus trees.

On UTUA 20, there was also significant change over the three sampling times, but here there was an evident contraction in the special positions occupied by *W. auropunctata* (Figure 6). Most interesting, there seems to be a relationship between the “young” colonies of *S. invicta* and the contraction of the *W. auropunctata*, especially between 2018 and 2019. In contrast, the change from July 2019 to January 2020 appears to have allowed *W. auropunctata* to reoccupy some of the space it seems to have lost to young *S. invicta* colonies the previous 6 months, perhaps suggesting a seasonal effect influencing the basic competitive interactions. Also notable is the reduction in occupation of old *S. invicta* colonies in the lower part of the plot over the 12 month period. In Table 3, we display the number of coffee bushes in UTUA 20 for each category of occurrence or co‐occurrence. It is clear that *W. auropunctata* co‐occurs much more frequently with young colonies of *S. invicta* (51% and 59%) than with old ones (0.5% and 3%), consistent with the hypothesis that *S. invicta* replaces *W. auropunctata*, at least temporarily. This replacement is hindered by the attacks of phorids, some species of which clearly prefer the megaworkers of *S. invicta*, which are abundant only in the older colonies. Thus, the young colonies of *S. invicta* (with few or no megaworkers) can dominate in competition with *W. auropunctata*, but as they become old (i.e., begin producing more megaworkers), the phorids become more active and abundant, thus detracting from the competitive dominance, and potentially reversing it to favor *W. auropunctata*.

**Table 3:** Co‐occurrence of W. auropunctata with S. invicta on the UTUA 20 farm on the three sampling dates (January 2019 (covering a sampling period from December 2,918 to January 2020), July 2019, and January 2020)

Species	January 19	July 19	January 20	Total
*Wasmannia auropunctata*	149	70	219	438
*Solenopsis invicta* (young nest)	111	170	149	430
*Solenopsis invicta* (old nest)	81	53	52	186
*S. invicta* (young nest) and *W. auropunctata*	67	109	78	254
*S. invicta* (old nest) and *W. auropunctata*	1	5	6	12

Note

Young swarms (i.e., presumably coming from young nests) are defined as those having few or no large majors while old swarms (i.e., presumably coming from older nests) are defined as those having significant numbers of large majors. Numbers are the number of coffee plants with the indicated ant presence.

### 3.3 Laboratory nest box trials of interactions between *W. auropunctata* and *S. invicta*

In the laboratory nest box trials, after connecting the nests, it became evident within hours that the *W. auropunctata* were severely affected by the foraging *S. invicta* workers. The inside walls of the nest boxes contained hundreds of *W. auropunctata* workers apparently trying to escape, and *S. invicta* workers were actively foraging in areas that had been occupied by *W. auropunctata*. Much of the *W. auropunctata* nesting material was woody stems with entrance holes small enough that *S. invicta* workers could not likely penetrate, so initial observations could not determine if the *W. auropunctata* workers were within those stems or not. A week after initiation of trials all *W. auropunctata* workers had disappeared, and nest boxes that had contained *W. auropunctata* were now occupied with *S. invicta* workers. Opening all woody stems that had been in the *W. auropunctata* nest boxes revealed a complete lack of *W. auropunctata* workers.

## 4 DISCUSSION

The ant assembly of this arboreally foraging ant community in this study is a dramatic example of a novel ecosystem in which we might expect clear ecological modalities to emerge (Perfecto & Vandermeer, 2015). Perhaps adding extra novelty is the fact that the most common of the species in the system are well‐known invasive species. If the expected modality forged by an “invasive” is the practical exclusion of other species, as is commonly thought, what emerges when the collection is mainly composed of such species? Perhaps the novelty here is simply alternative states of single species dominance in a large area, perhaps generating an unusual form of a metacommunity at a very large scale. We see some farms that, at least for a 12 month period, retain the dominance of a single one of these invasive species, while the change from one farm to another suggests that the permanent monospecific dominance is necessarily temporary, at least at a local level.

At a macro‐scale (25 farms across the entire coffee‐growing region of Puerto Rico; Figure 1), there is a great deal of variability in this novel community (Table 1). Although the majority of farms retained the main species dominance over the 12 month sampling period, several had major transformations, including five cases in which the site contained a species that had not been there on the previous sampling date (Table 2). We suspect that a 10 × 10 m^2^ sampling plot did not really sample the biodiversity on the farm as a whole, as evidenced by the more extensive sampling on the two intensively sampled farms (Figures 5 and 6). While the classification of UTUA 2 as a *W. auropunctata* farm was accurate, the classification of UTUA 20 as a *S. invicta* farm was completely misleading (Figure 6).

Regarding *S. invicta*, the notable difference between the swarms identified as coming from young colonies and those coming from older colonies and the relationship thereof with *W. auropunctata* (Table 3) defies any direct and obvious interpretation. The pattern could be related to the abundant phorid fly parasitoids (*Pseudacteon* spp.) which we regularly observe on swarms of *S. invicta* on the ground (rarely on the arboreal baits). It is well‐established that phorids have a dramatic effect on the ecology of *S. invicta* (e.g., Chirino, Gilbert, & Folgarait, 2009; Morrison, 1999; Morrison & Porter, 2005; Puckett & Harris, 2010; Reed, Puckett, & Gold, 2015). It is evident that at least the most commonly observed phorid species has a very strong preference for the larger majors in a swarm of old *S. invicta*. We hypothesize that the harassment from these flies interferes with the foraging ability of workers from the older colonies more than the younger ones and makes the older colonies less competitive with *W. auropunctata*. Studies of the effect of phorid flies on size ratios of *S. invicta* foragers, document an increase of small foragers in the presence of phorid flies in both native and introduce habitats of *S. invicta* (Chirino et al., 2009; Puckett & Harris, 2010; Reed et al., 2015). The harassment effect of the phorid flies can also affect competitive interactions between species (Morrison, 1999). In our study, nonsystematic but extensive observations on the behavior of the phorid flies suggest they may have a very large effect. For example, in one case a single phorid was seen to attack at least 10 and perhaps as many as 20 workers in a one minute observation period. Multiplying that number by the number of hours available for phorid attack, and the potential effect on workers could be substantial. However, we should also note that in laboratory experiments as well as an extensive three‐year field study of the effect of an introduced phorid species on *S. invicta* in Florida, the authors failed to find an effect of parasitism pressure on density or activity of *S. invicta* (Morrison & Porter, 2005; Mottern, Heinz, & Ode, 2004). Whatever the mechanism, it is evident that there is a significant change in the pattern of occurrence across the 12 month sampling period on UTUA 2 and UTUA 20 with respect to *S. invicta* and *W. auropunctata* (Figures 5 and 6).

On farm UTUA2, there are two qualitative patterns that stand out (Figure 5). In January 2019, *W. auropunctata* clearly dominated most of the area, but was relatively rare on the right hand part of the sampling area. By July 2019, *S. invicta* had increased its activity significantly on the right part of the plot, with coffee bushes mainly harboring old colonies, presumably excellent targets for the phorids. In January 2020, there were two evident events that emerged. First, the concentration of older colonies that had been on the right part of the plot in July 2019 disappeared almost entirely, perhaps due to large‐scale attack from phorids. Second, a group of coffee bushes were recorded to be occupied by foraging swarms from young colonies of *S. invicta* in the middle of the area formerly dominated by *W. auropunctata*. Furthermore, activity on the ground of *S. invicta* was considerably more extensive than the activity on the bushes themselves, suggesting that we are witnessing a local “invasion” of *S. invicta*, perhaps a single colony. In searching the ground for surface mounds, only a single very small mound was encountered immediately at the edge of the area that *S. invicta* was invading.

On farm UTUA 20, from January to July 2019, there was a dramatic increase in the number and extent of *S. invicta* foragers from young colonies, accompanied by a reduction in bushes occupied by *W. auropunctata* (Figure 6 and Table 3). Furthermore, the pattern of occurrence on the farm was clearly not random, with the distribution of *W. auropunctata* seemingly restricted from both above and below by the incursion of *S. invicta*. This pattern was slightly reversed between 2019 and 2020, perhaps reflecting a seasonal component of the dynamics. Also, the concentration of *S. invicta* old colonies near the lower right of the plot was dramatically reduced by July 2019, consistent with the idea of a phorid effect on older colonies. Casual observations regularly observed phorids attacking *S. invicta* in this area.

**Figure 7:**
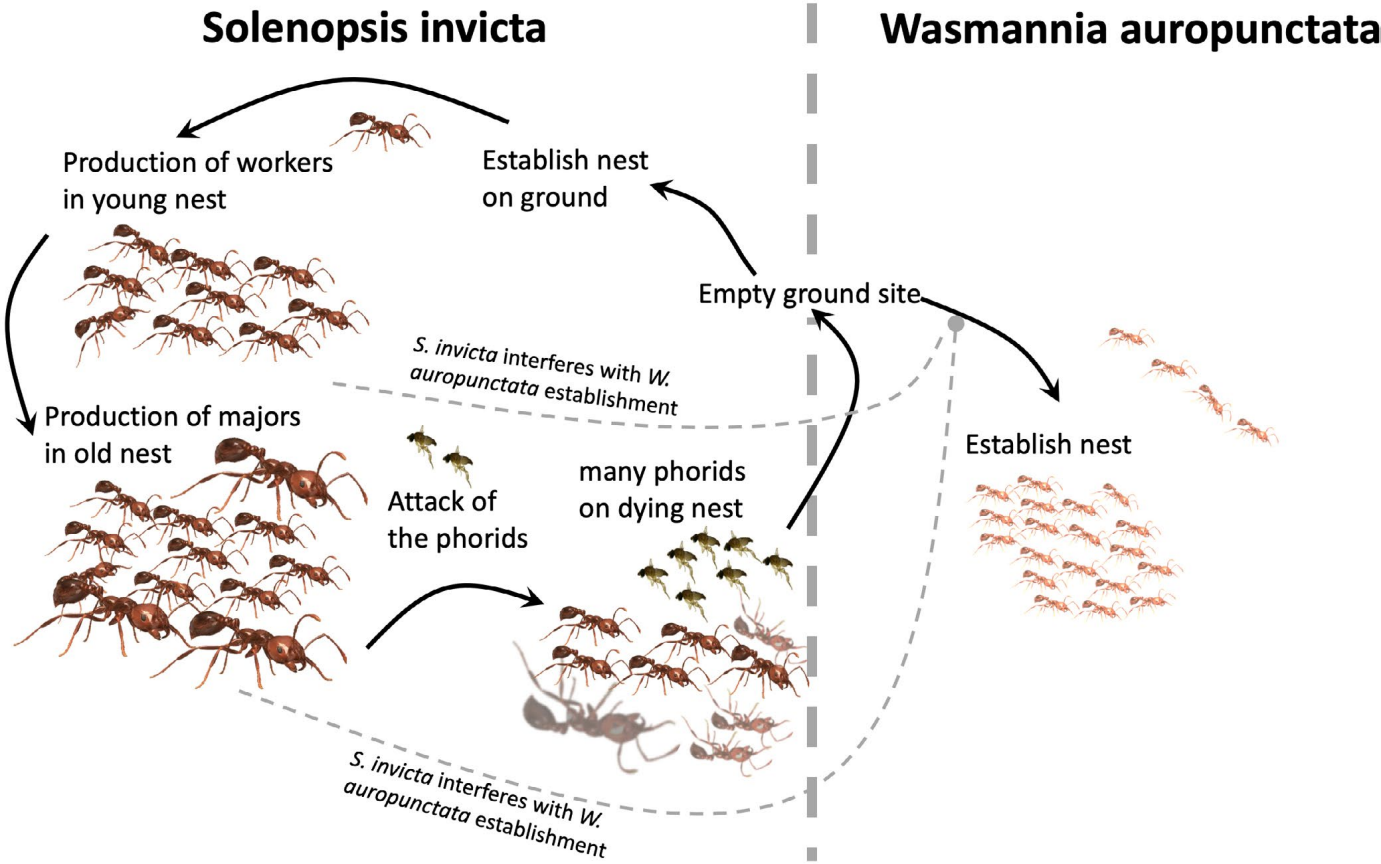
Diagrammatic picture of the hypothetical spatio/temporal competitive process between *S. invicta* and *W. auropunctata*. Dashed connections indicate the negative effect that *S. invicta* colonies are hypothesized to have on the establishment and/or survival of *W. auropunctata* nests

Given these general spatial and temporal patterns, combined with the abundant literature documenting the importance of phorid flies on *Solenopsis* species (Chen & Fadamiro, 2018; Feener & Brown, 1992; Oi et al., 2019; Porter, 1998; Porter, Meer, Pesquero, Campiolo, & Fowler, 1995; Puckett & Harris, 2010), it is possible to suggest a narrative of how *S. invicta* and *W. auropunctata* interact in the coffee‐growing region of Puerto Rico. When a colony enters an “empty” space, either from a founding queen or a queen moving with some of her workers and brood, it persists there when normal resources are available. Eventually, a colony from the other species co‐occupies the space, challenging the first species for available resources. Notably, both species actively tend scale insects and other hemipterans on coffee trees, prey on other insects, and scavenge for organic detritus both on the trees and on the ground below, and thus are likely to compete, at least over the long term (Torres, 1984b). When the occupying colony is *S. invicta*, its foraging advantage begins the process of competitively excluding *W. auropunctata* from the site. As *S. invicta* spreads locally to nearby coffee bushes, its population builds up to the point that it begins producing the mega‐workers so characteristic of older colonies (Tschinkel, 1988). As the numbers of mega‐workers continues increasing, the local phorid population begins to increase. Eventually, the phorids become so common that the *S. invicta* colony either dies or moves to a site considerably removed from the local concentration of phorids. This narrative is illustrated qualitatively in Figure 7. This sort of dynamic process of competition is both spatial and temporal and is a narrative that concords well with observations on both of the intensively studied farms as well as the more spatially extensive observations of this novel community over the entire coffee production area.

It is important to note that the process of competition suggested here is speculative since we do not have direct evidence of the competition between these two species. Although laboratory trials did demonstrate strong aggressive behavior of *S. invicta* workers against *W. auropunctata*, this type of antagonistic behavior between a pair of species does not necessarily imply interspecific competition, because competition is a population level process not and individual level process. Aggressive behavior is a component of competition in ants, to be sure, but as have been noted elsewhere (Perfecto, 1994), competitive outcomes can easily be the reverse of what aggressive encounters might imply. Additionally, we cannot infer competition from species distribution data alone since abiotic conditions, like nesting sites or food availability could be the structuring mechanisms (Parr & Gibb, 2010). However, the data that we accumulated do fit with the narrative in Figure 7. It will take more detailed and controlled experiments to test the proposed process that we speculate based on descriptive data and field observations.

These results are of practical significance since *W. auropunctata* is regarded as one of the most important “pests” in the coffee system due to its effect on harvesting efficiency (informal interviews with multiple coffee farmers). Yet, it has potential to be a major natural enemy of at least two of the major pests in coffee, the coffee leaf miner, *Leucoptera coffeella* (Perfecto & Vandermeer, unpublished data) and the coffee berry borer *Hypothenemus hampei* (Morris et al., 2018). Elsewhere, we report on the complicated antagonistic relationship between *W. auropunctata* and lizards of the genus Anolis (), the latter of which appear to be significant natural enemies of both the coffee berry borer (Monagan, Morris, Davis Rabosky, Perfecto, & Vandermeer, 2017) and the miner (Perfecto, Hajian‐Forooshani, White, & Vandermeer, 2021). The end result may be that the potential biological control effect of *W. auropunctata* is countered by its negative effect on the more efficient controlling agents, the anoline lizards. Understanding the effect of other ant species on this noxious ant may aid in developing strategies to limit its presence. In this study, *W. auropunctata* dominated only six of the 25 farms surveyed, suggesting that its notoriety as one of the most important pests in the system is hardly ubiquitous. However, in the farms where it is present, it is certainly a problem for farmers, particularly during the harvest period. Understanding the forces that make it dominant on some farms while virtually absent on others may lead to strategies for managing it.

## CONFLICT OF INTEREST

The authors declare no competing interests.

## AUTHOR CONTRIBUTION

**Ivette Perfecto:** Conceptualization (equal); Data curation (supporting); Formal analysis (equal); Funding acquisition (lead); Investigation (equal); Methodology (equal); Project administration (lead); Resources (equal); Visualization (equal); Writing‐original draft (equal); Writing‐review & editing (supporting). **John Vandermeer:** Conceptualization (equal); Data curation (lead); Formal analysis (lead); Funding acquisition (supporting); Investigation (equal); Methodology (equal); Project administration (supporting); Resources (equal); Visualization (equal); Writing‐original draft (supporting); Writing‐review & editing (lead).

## Supporting information

TableS1

## Data Availability

Data for this study are available inn Dryad under "Data Base for novel ant ecosystems" https://doi.org/10.5061/dryad.8sf7m0ck5
