# Supplementary material for: The assembly and importance of a novel ecosystem: The ant community of coffee farms in Puerto Rico
Source: Ecol Evol. 2020 Oct 27;10(23):12650–62. doi: 10.1002/ece3.6785 (PMC7713940; doi:10.1002/ece3.6785)
Supplement: Supplementary file 1 — TableS1 [file ECE3-10-12650-s001.docx]

Table S1: Location, elevation and percent canopy cover for each farm in the study.

| Farm code | latitude | longitude | elevation | % canopy cover |
| --- | --- | --- | --- | --- |
| ADJU7 | 18.196703 | -66.831782 | 464.15 | 0.00 |
| ADJU8 | 18.229713 | -66.806609 | 691.44 | 0.00 |
| JAYU2 | 18.216102 | -66.567247 | 550.31 | 6.26 |
| JAYU3 | 18.212975 | -66.564589 | 524.36 | 14.97 |
| JUAN1 | 18.129795 | -66.531003 | 626.40 | 16.10 |
| JUAN7 | 18.119929 | -66.558415 | 452.43 | 0.00 |
| LASM1 | 18.222208 | -67.000595 | 344.21 | 8.06 |
| LASM2 | 18.213724 | -66.975687 | 411.40 | 0.00 |
| LASM3 | 18.209639 | -66.979641 | 447.86 | 56.98 |
| MARI2 | 18.154216 | -66.934898 | 652.87 | 36.42 |
| MARI3 | 18.152315 | -66.930338 | 725.31 | 5.70 |
| MARI8 | 18.152028 | -66.892094 | 882.31 | 35.22 |
| OROC1 | 18.175301 | -66.416165 | 751.70 | 47.30 |
| PONC | 18.114617 | -66.558387 | 530.93 | 17.30 |
| UTUA10 | 18.263964 | -66.611003 | 659.19 | 7.43 |
| UTUA13 | 18.262440 | -66.784717 | 542.75 | 2.58 |
| UTUA16 | 18.271612 | -66.670071 | 507.47 | 17.32 |
| UTUA17 | 18.269363 | -66.742064 | 530.26 | 2.45 |
| UTUA18 | 18.272386 | -66.754348 | 525.30 | 0.63 |
| UTUA2 | 18.287858 | -66.770264 | 394.81 | 25.76 |
| UTUA20 | 18.279491 | -66.828209 | 429.72 | 0.00 |
| UTUA30 | 18.260127 | -66.605766 | 648.54 | 0.00 |
| UTUA5 | 18.261009 | -66.618559 | 727.56 | 19.73 |
| YAUC3 | 18.144023 | -66.819158 | 887.25 | 26.92 |
| YAUC4 | 18.149636 | -66.842446 | 776.39 | 37.11 |
